# Development and Validation of a Multidisciplinary Mobile Care System for Patients With Advanced Gastrointestinal Cancer: Interventional Observation Study

**DOI:** 10.2196/mhealth.9363

**Published:** 2018-05-07

**Authors:** Ji Yeong Soh, Won Chul Cha, Dong Kyung Chang, Ji Hye Hwang, Kihyung Kim, Miyong Rha, Hee Kwon

**Affiliations:** ^1^ Samsung Advanced Institute for Health Sciences & Technology Department of Digital Health Sungkyunkwan University Seoul Republic Of Korea; ^2^ Department of Physical and Rehabilitation Medicine Sungkyunkwan University Seoul Republic Of Korea; ^3^ Department of Dietetics Samsung Medical Center Seoul Republic Of Korea; ^4^ LifeSemantics Corp Seoul Republic Of Korea

**Keywords:** mobile health, health apps, mobile phone, mobile care system

## Abstract

**Background:**

Mobile health apps have emerged as supportive tools in the management of advanced cancers. However, only a few apps have self-monitoring features, and they are not standardized and validated.

**Objective:**

This study aimed to develop and validate a multidisciplinary mobile care system with self-monitoring features that can be useful for patients with advanced gastrointestinal cancer.

**Methods:**

The development of the multidisciplinary mobile health management system was divided into 3 steps. First, the service scope was set up, and the measurement tools were standardized. Second, the service flow of the mobile care system was organized. Third, the mobile app (Life Manager) was developed. The app was developed to achieve 3 major clinical goals: support for quality of life, nutrition, and rehabilitation. Three main functional themes were developed to achieve clinical goals: a to-do list, health education, and in-app chat. Thirteen clinically oriented measures were included: the modified Patient-Reported Outcomes version of the Common Terminology Criteria for Adverse Events questionnaire, Scored Patient-Generated Subjective Global Assessment (PG-SGA), distress, European Organization for Research and Treatment of Cancer Quality of Life Questionnaire, International Physical Activity Questionnaire–Short Form, Low anterior resection syndrome score, satisfaction rate, etc. To validate the system, a prospective observational study was conducted. Patients with gastric cancer or colon cancer undergoing chemotherapy were recruited. We followed the subjects for 12 weeks, and selected clinical measures were taken online and offline.

**Results:**

After the development process, a multidisciplinary app, the Life Manager, was launched. For evaluation, 203 patients were recruited for the study, of whom 101 (49.8%) had gastric cancer, and 102 (50.2%) were receiving palliative care. Most patients were in their fifties (35.5%), and 128 (63.1%) were male. Overall, 176 subjects (86.7%) completed the study. Among subjects who dropped out, the most common reason was the change of patient’s clinical condition (51.9%). During the study period, subjects received multiple health education sessions. For the gastric cancer group, the “general gastric cancer education” was most frequently viewed (322 times), and for the colon cancer group, the “warming-up exercise” was most viewed (340 times). Of 13 measurements taken from subjects, 9 were taken offline (response rate: 52.0% to 90.1%), and 3 were taken online (response rate: 17.6% to 57.4%). The overall satisfaction rate among subjects was favorable and ranged from 3.93 (SD 0.88) to 4.01 (SD 0.87) on the 5-point Likert scale.

**Conclusions:**

A multidisciplinary mobile care system for patients with advanced gastrointestinal cancer was developed with clinically oriented measures. A prospective study was performed for its evaluation, which showed favorable satisfaction.

## Introduction

Cancer is a major cause of death worldwide. It is one of the leading causes of morbidity and mortality with approximately 14 million new cases in 2012. Moreover, cancer accounted for 8.8 million deaths in 2015 and is the number 1 cause of death in Korea [1]. Although the survival rate of cancer has increased due to the advancement of diagnostic and therapeutic modalities, the majority of patients still suffer from numerous physical, psychological, and social difficulties [2,3]. Chemotherapy is the most common treatment modality for patients with advanced cancer. Although these treatments can improve the survival of patients, quality of life remains poor because of the adverse effects of the treatments [4-6].

Lifestyle modification, good nutritional status, and appropriate exercise are extremely important because they mitigate treatment effects and the morbidity, mortality, and quality of life of patients [7]. However, most patients fail to acquire sufficient information that is applicable to daily living [8]. Moreover, patients rarely use tools to report subjective information such as pain, fatigue, anorexia, and distress [9,10].

Mobile health apps have emerged as supportive tools in the management of cancer. A well-established health app can be beneficial for patients with cancer because it reduces financial burden, provides access to information, and facilitates communication [11-13]. However, only a few apps have self-monitoring features, and they often lack standardized validation in terms of benefits, acceptance, costs, and risks [14-16]. In order to set up a clinically validated service, a multidisciplinary team of health care experts must be involved in all stages of the design of the app architecture [17].

This study aimed to develop and validate a multidisciplinary mobile care system that can provide health education and self-management features to improve multiple clinical measures for patients with advanced gastrointestinal cancer.

## Methods

### Overview

This study comprised a development phase (May 2016 to October 2017) and a validation phase (September 2016 to December 2017). The study was approved by the institutional review board of the study site (2016-05-010).

### System Development

In this study, the establishment of a mobile health management system comprised 3 steps: (1) establishment of the service scope and standardization of the measurement tools, (2) organization of service process, and (3) development of the mobile app (Life Manager).

### Establishment of the Service Scope and Standardization of the Measurement Tools

For the preparation of the multidisciplinary mobile care system and service flow, scope of service was established, and the health care users were identified. Health care professionals (N=13) were recruited from a comprehensive cancer center in Seoul. Medical professionals including 6 gastrointestinal oncologists, a specialist oncology nurse, 2 oncology rehabilitation physicians, 2 nutrition specialists, a cancer education specialist, and a customer relationship manager expert joined the team. In multiple rounds of meetings, the team agreed on the final selection of clinically measurable outcomes with 3 major clinical goals: quality of life, supporting rehabilitation, and improving nutritional state (Table 1).

In addition, we reviewed nutritional assessment tools such as the Malnutrition Universal Screening Tool and the Academy of Nutrition and Dietetics/American Society for Parenteral and Enteral Nutrition Consensus Statement [18-20] and quality of life tools such as the Medical Outcome Study Short Form–36 and EuroQol-5 dimension [20,21]. The following measures were consequently selected based on their feasibility and reliability.

For online measurements, the Patient-Reported Outcomes version of the Common Terminology Criteria for Adverse Events (PRO-CTCAE) [22-24], nutritional survey, and rehabilitation survey were collated. The Common Terminology Criteria for Adverse Events (CTCAE) is maintained by the US National Cancer Institute. The CTCAE for each item represents a discrete event that is graded for severity on a 5-point scale based on clinical criteria.

For offline measurements, 9 indices were included. For quality of life, the European Organization for Research and Treatment of Cancer Quality of Life Questionnaire (EORTC QLQ-30) was included. It was developed to assess the health-related quality of life of cancer patients and has been validated in various studies. Distress is a frequent symptom patients suffer from during their journey with cancer and has been a major focus recently, and thus distress was included as a quality measurement [25,26]. For the nutritional goal, the Scored Patient-Generated Subjective Global Assessment (PG-SGA) was included. It is already used internationally as a reference method for proactive risk assessment (screening), assessment, monitoring, and triaging for interventions in patients with cancer [27]. For rehabilitation, the International Physical Activity Questionnaire–Short Form (IPAQ-SF), Low Anterior Resection Syndrome (LARS) score, and Brief Fatigue Inventory–Korean (BFI-K) were chosen as measurements [28-32].

### Organization of Service Process

To complete the multidisciplinary mobile care system, the service protocol must be clearly defined. The service protocol consists of an offline and online protocol. The service protocol consists of offline care flow (face-to-face–based care flow) and online care flow (mobile-based care flow). Our primary goal in the clinical service protocol is to create an optimal mobile health demonstration model that can be followed by anyone.

**Figure 1:**
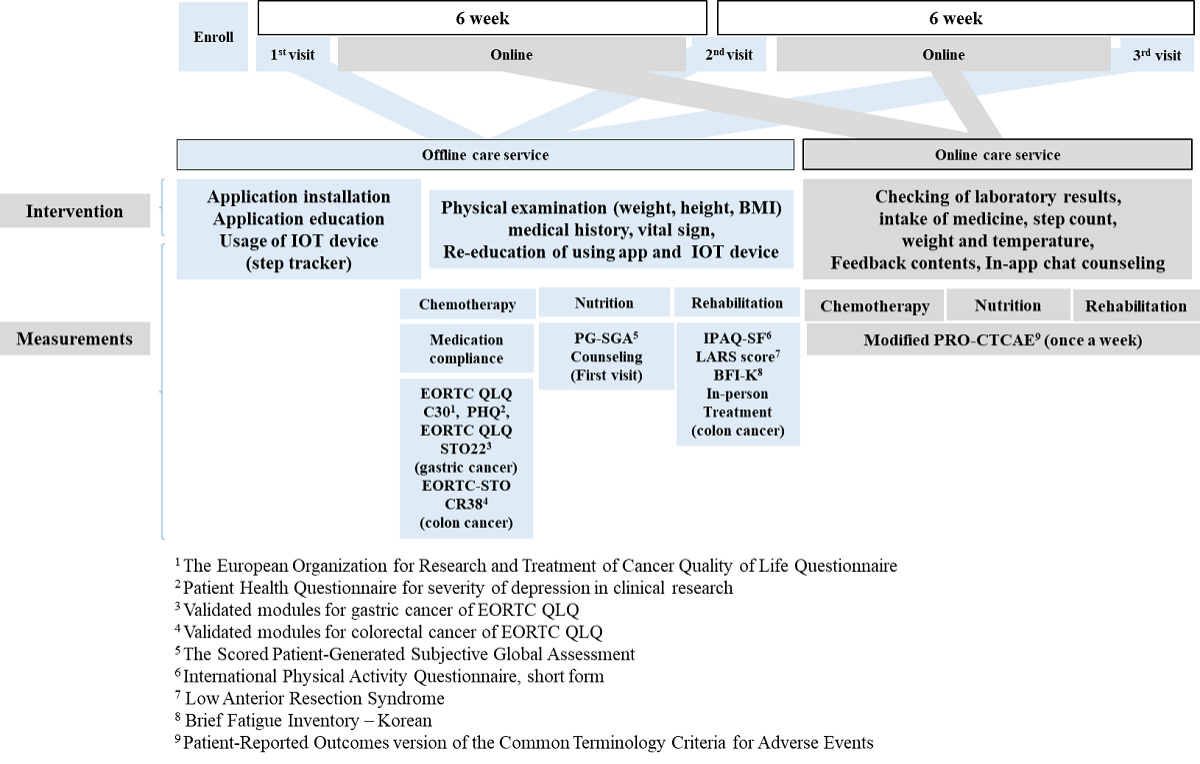
User service diagram. Patients with advanced gastrointestinal cancer are able to use this care service for 12 weeks. The user can receive comprehensive care service from health providers (medical doctors, nurses, nutritionists, and rehabilitation specialists).

The offline service flow was designed not to interfere with the preexisting clinical process. The online service was carefully organized so that the same treatment goal can be achieved with the offline service. To date, the service flow of the mobile care system with clinical basis has been meticulously established based on literature review and expert opinion (Figure 1).

**Table 1:** Measurements of clinical outcome and system performance of the Life Manager.

Goals	Online measurements	Offline measurements
Quality of life	Modified PRO-CTCAE^a^	EORTC QLQ-STO22^b^ (gastric)
	—	EORTC QLQ-CR38^c^ (colon)
	—	EORTC QLQ-30^d^
	—	Distress
Nutrition	Nutrition survey	Scored Patient-Generated Subjective Global Assessment
Rehabilitation	Rehabilitation survey	International Physical Activity Questionnaire–Short Form (gastric)
	—	Low Anterior Resection Syndrome score (colon)
	—	Brief Fatigue Inventory–Korean
Satisfaction	—	Satisfaction survey

^a^PRO-CTCAE: Patient-Reported Outcomes version of the Common Terminology Criteria for Adverse Events.

^b^EORTC QLQ-STO22: European Organization for Research and Treatment of Cancer Quality of Life Questionnaire–Gastric module.

^c^EORTC QLQ-CR38: European Organization for Research and Treatment of Cancer Quality of Life Questionnaire–Colorectal module.

^d^EORTC QLQ-30: European Organization for Research and Treatment of Cancer Quality of Life Questionnaire.

### Development of the Mobile App (Life Manager)

#### System Architecture

The core function of the mobile health app was established by gathering opinions from various health care professionals. Technicians and developers designed an architecture model to functionally support those main themes selected by the clinical part members.

**Figure 2:**
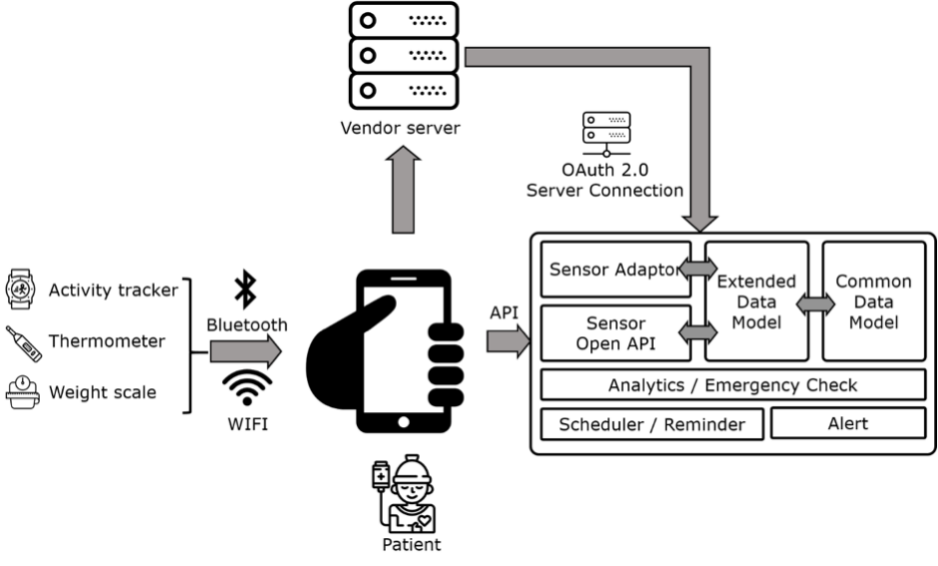
System architecture of Life Manager. Data from patients were collected through the app and processed with predesigned rules. API: application programming interface.

The number of steps through the activity tracker, body temperature, and weight are collected in the patient app via Wi-Fi, Bluetooth, and manual input. The collected information was recorded automatically and checked on the administrator webpage. It was used by health providers to design clinical interventions. For security, personal information was stored as unidentified code, and the OAuth 2.0 protocol, a standardized security method, was used [7] (Figure 2).

#### Designing the User Interface

**Figure 3:**
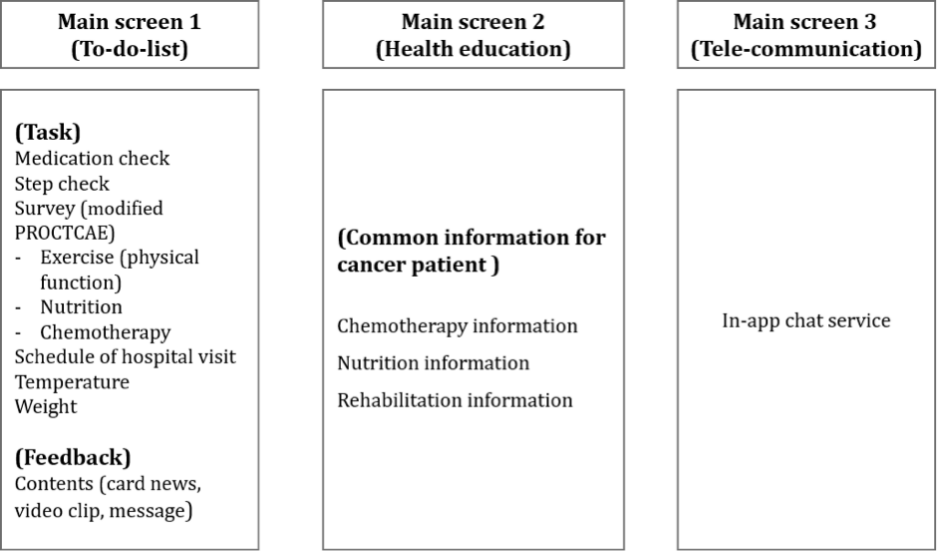
General concept of the Life Manager. The app has 3 main screens. Screen 1 shows the To-Do list theme, screen 2 depicts the Health education theme, and screen 3 shows the Telecommunication (In-app chat) theme. PRO-CTCAE: Patient-Reported Outcomes version of the Common Terminology Criteria for Adverse Events.

The main themes, including the core function of the mobile app, aimed to maintain the quality of care even if the patient is outside of the hospital. It uses a standard health domain to provide a high level of clinical evidence based on the services provided by various clinical professionals. The mobile app comprised 3 main themes. Figure 3 shows the main functions implemented on each screen.

#### The Final App

Three main application themes were used. First, the To-Do list theme was used. When the patient installs the Life Manager app and logs in, they first see the To-Do list screen. The patient can check the Daily tasks on this screen. The user can see the core function of the mobile questionnaire (PRO-CTCAE) and feedback contents; check the medications to be taken, achievement in walking exercise, and schedule of a hospital visit in the screen; and measure temperature and weight. When the patient completes the daily task, the color of the task screen changes to confirm the achievement rate of the patient.

**Figure 4:**
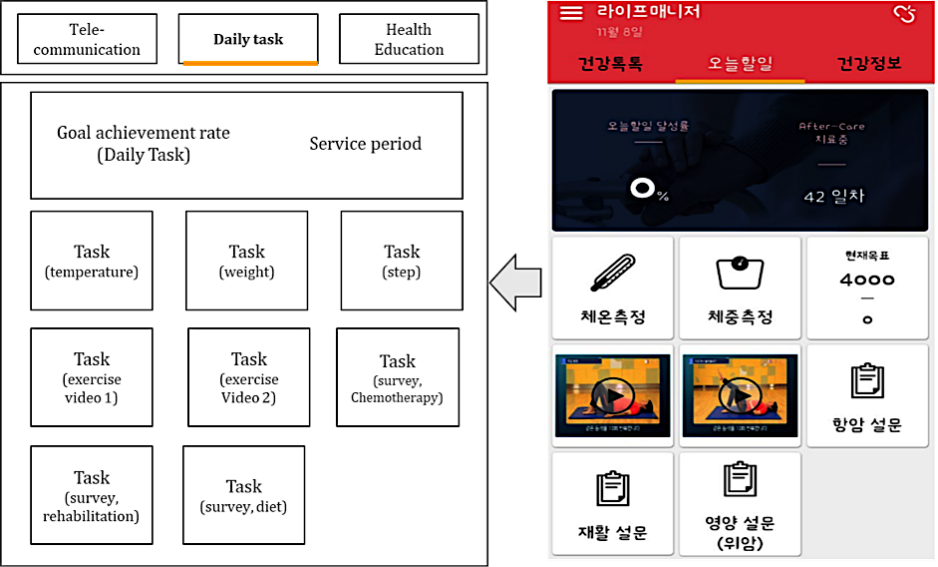
Main screen of the Life Manager app. Image on the right is an actual screen capture from the app, while image on the left is its translation to English. After patients log in to the app, they can first see the To-Do list screen. Patients can check the Daily tasks on this screen, which can be changed every day depending on the condition of the users.

Second, the Health education theme was used, which addresses common questions that patients have. The common contents of this theme include drug information, general side effects, and countermeasures against the side effects of chemotherapy. Third, the In-app chat service theme was used, which can facilitate communication with experts anytime and anywhere (Figure 4).

Activity information is measured through the wearable device that is linked to the app via Bluetooth. The step counts and calorie expenditure are recorded in real time. The log file is presented in the form of a statistical graph in the management system, and the patient’s health record can be used for checking using the Life Manager app. Health data that are automatically collected from the wearable device and health data that are manually entered by an individual are recorded from the Life Manager’s server to its platform. Collection and life log are encrypted by the Life Manager platform and then processed for transmission.

#### Validation of the System

##### Study Design and Setting

We carried out a single-center prospective descriptive study to validate the system. The site of the study was a tertiary academic center with 2000 inpatient beds. There are approximately 8000 outpatient visits and 200 emergency visits per day.

##### Study Participants

Patients with gastric cancer and colon cancer were the main subjects of the study. The inclusion criteria were as follows: colon or gastric cancer diagnosis, underwent surgery for the cancer diagnosis, receiving chemotherapy for the cancer, have an Android mobile phone version 4.3 or higher, aged older than 18 years, and consented to participate in the study.

The exclusion criteria were as follows: not eligible for offline follow-up (eg, home very far away), active Do Not Attempt Resuscitation order, confusion or altered mental status, and could not follow the instructions of the study coordinators at the initial demonstration.

### Outcome and Measurements

The primary outcome was systemic measurement of subject satisfaction. The secondary outcome was response rate to the clinical measurements described in Table 1. Subject demographic data along with information regarding disease status and treatment plan were also gathered.

### Statistical Analysis

A simple descriptive analysis was performed to observe the outcome. The outcome was described based on patient cancer types.

## Results

### Characteristics of Study Participants

A total of 203 participants were recruited for the study, 101 and 102 of whom had gastric and colon cancer, respectively. There were more males than females. While 55.4% (56/101) of gastric cancer patients were receiving adjuvant therapy, 44.6% (45/101) were on palliative treatment. For colon cancer, the adjuvant group comprised 44.1% (45/102), and the palliative group comprised 55.9% (57/102; Table 2).

### Participant Completion Rate and Response Rate for Each Measurement

Completion was defined if a subject could respond to all of the offline surveys. Overall, 176 out of 203 (86.7%) subjects completed the program successfully. The most common reason for dropout was change in physical condition of subject, followed by difficulty of app use (Table 3).

Tables 4 and 5 show the response rates of subjects for each measurement. For the offline surveys, the response rate was relatively high excluding early dropouts. Measurements from the third visit were lower than those from the second. Surveys for medication compliance showed the lowest response rate. The online surveys were individualized according to subjects’ clinical settings. The response rate ranged from 17.6% to 57.4%.

### Health Education Content Views

For health education, a total of 2338 contents were viewed by the gastric cancer group and 3071 by the colon cancer group. The overall frequency of views is described in Table 6. For gastric cancer, the most commonly viewed content was “gastric cancer general information” (322 times), and for colon cancer “warming up exercise” was viewed 340 times (Table 6).

### Satisfaction Rate

**Figure 5:**
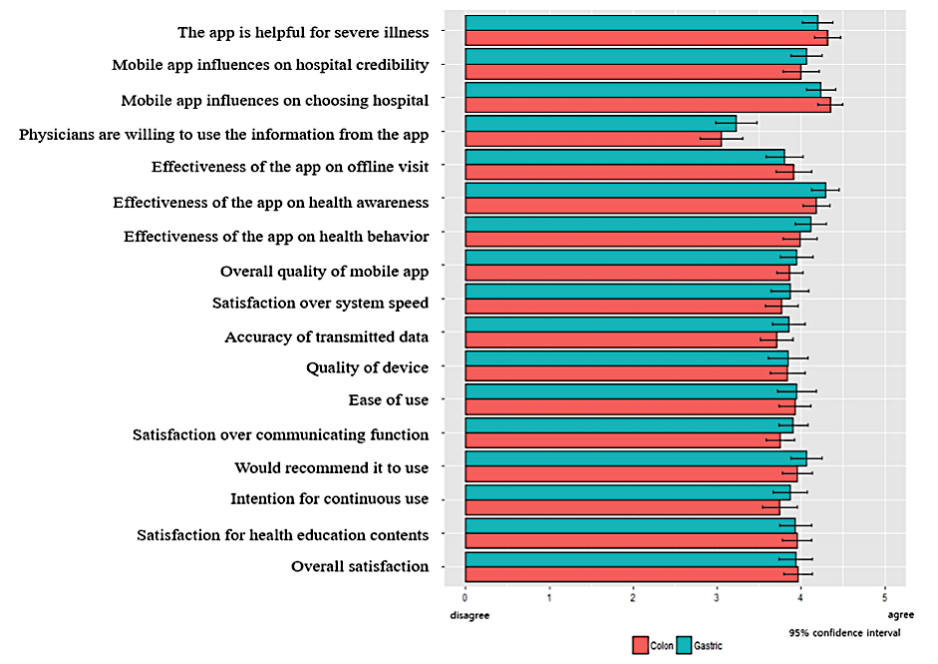
Satisfaction rate of study participants.

The satisfaction rate was measured on a 5-point Likert scale (5=very good, 4=good, 3=neutral, 2=bad, 1=very bad). The most valued components were “appropriateness to management” and “continuous visit to this hospital.” The lowest satisfaction rate was seen in “this program assists the medical doctor” (Figure 5).

**Table 2:** Demographic information of study participants.

Characteristics		Gastric cancer (n=101), n (%)	Colon cancer (n=102), n (%)
**Sex**			
	Male	71 (70.3)	57 (55.9)
	Female	30 (29.7)	45 (44.1)
**Age, years**			
	Less than 40	12 (11.9)	4 (3.9)
	40s	24 (23.8)	15 (14.7)
	50s	37 (36.6)	35 (34.3)
	60s	22 (21.8)	35 (34.3)
	Over 70	6 (5.9)	13 (12.7)
**Treatment plan**			
	Adjuvant	56 (55.4)	45 (44.1)
	Palliative	45 (44.6)	57 (55.9)

**Table 3:** Study completion rates of study subjects.

Characteristics			Gastric cancer (n=101), n (%)		Colon cancern (n=102), n (%)
Subjects with successful completion			85 (84.2)		91 (89.2)
**Subjects who dropped out**			16 (15.8)		11(10.8)
	Physical condition change	8 (50.0)		6 (54.5)	
	Difficulty manipulating the app	5 (31.3)		4 (36.4)	
	Transfer to other hospital	2 (12.5)		1 (9.1)	
	Other reason	1 (6.3)		0 (0)	

**Table 4:** Response rate for the offline surveys.

Task	Gastric cancer (n=101), n (%)		Colon cancer (n=102), n (%)	
	2^nd^ visit	3^rd^ visit	2^nd^ visit	3^rd^ visit
Medication compliance	90 (89.1)	78 (77.2)	66 (64.7)	53 (52.0)
PG-SGA^a^	90 (89.1)	85 (84.2)	91 (89.2)	88 (86.3)
IPAQ-SF^b^	90 (89.1)	85 (84.2)	91 (89.2)	89 (87.3)
BFI-K^c^	90 (89.1)	85 (84.2)	—	90 (88.2)
LARS^d^ score	—	—	92 (90.2)	89 (87.3)
EORTC QLQ-C30^e^	90 (89.1)	85 (84.2)	92 (90.2)	89 (87.3)
EORTC QLQ-STO22^f^	90 (89.1)	85 (84.2)	—	—
EORTC QLQ-CR38^g^	—	—	92 (90.2)	89 (87.3)
PHQ-9^h^	91 (90.1)	85 (84.2)	92 (90.2)	89 (87.3)
Distress	90 (89.1)	85 (84.2)	91 (89.2)	88 (86.3)

^a^PG-SGA: Scored Patient-Generated Subjective Global Assessment.

^b^IPAQ-SF: International Physical Activity Questionnaire–Short Form.

^c^BFI-K: Brief Fatigue Inventory–Korean.

^d^LARS: Low Anterior Resection Syndrome.

^e^EORTC QLQ-30: European Organization for Research and Treatment of Cancer Quality of Life Questionnaire.

^f^EORTC QLQ-STO22: European Organization for Research and Treatment of Cancer Quality of Life Questionnaire–Gastric module.

^g^EORTC QLQ-CR38: European Organization for Research and Treatment of Cancer Quality of Life Questionnaire–Colorectal module.

^h^PHQ-9: Patient Health Questionnaire–9 item.

**Table 5:** Response rate for online surveys.

Online survey category	Gastric cancer		Colon cancer	
	Sent, n	Response, n (%)	Sent, n	Response, n (%)
Modified PRO-CTCAE^a^	940	502 (53.4)	1000	527 (52.7)
Nutrition	820	471 (57.4)	854	150 (17.6)
Rehabilitation	756	343 (45.4)	794	387 (48.7)
Total	2516	1316 (52.3)	26,481	1064 (40.2)

^a^PRO-CTCAE: Patient-Reported Outcomes version of the Common Terminology Criteria for Adverse Events.

**Table 6:** Health education content views by participants.

Rank	Gastric cancer (n=2338)	Views	Colon cancer (n=3071)	Views
1	Cancer general information	322	Warming-up exercise	340
2	Warming-up exercise	284	Muscle strength exercise 2	320
3	Muscle strength exercise 1	275	Flexibility exercise	292
4	Flexibility exercise	255	Pelvic floor muscle exercise	260
5	Skin problem	76	Cancer general information 3	220
6	Sleep problem	67	Cancer general information 2	143
7	General exhaustion	59	Sleep problem	132
8	Hand and foot swelling	58	Hair loss	118
9	Constipation	53	Skin problem (clammy)	116
10	Skin problem (colorization)	44	Skin problem (dry)	115

## Discussion

### Development Process

Many mobile health apps have been developed for cancer patients, although it was not easy to find apps that apply personalized interventions for nutrition, rehabilitation, and side effects in patients who are undergoing chemotherapy. Only a few of such apps showed details of how nutritionists, rehabilitation therapists, cancer nurses, doctors, and various health providers intervened when providing personalized intervention through apps and communicating with patients [33-35].

During this study, we successfully developed a multidisciplinary mobile care system that could provide health education and self-management features for clinical improvement. Measures were chosen based on clinical evidence by experts who were actively involved in the treatment process of patients. This is contrary to the fact that many apps developed for patients do not acquire data which could be readily used in real clinical settings [36].

Also, this program joined the processes of offline and online interventions, which is said to be an important factor impacting the success of mobile apps. Mobile app developers need to analyze the process of hospital-based care in order to effectively help patients. By recruiting caregivers of such a process, we could develop a study program which could infiltrate patient predefined treatment processes with minimal discomfort, resulting in a high completion rate.

### Validation Process

We enrolled 203 subjects from September 2016 to December 2016. The online survey completion rate was over 40%, and 80% completed the offline survey. The overall program completion rate was about 85%. Considering patients’ poor general conditions and older ages, these figures are somewhat encouraging [37]. Patient-oriented user interfaces with user-friendly services like in-app chat service could be reasons for good compliance.

Throughout the study, coordinators freely contacted participants using the app, providing chat service and preproduced educational content; they could also meet patients when they had arrangements in the outpatient department or during scheduled chemotherapy sessions. This environment could have affected the favorable outcome of clinical validation. However, continuity of care with the physician offices was not observed in this study, which was also revealed by in-depth satisfaction analysis (Table 5).

### Summary of the Implications of the Research

First, the mobile care system enabled patients with severe conditions to obtain personalized health management and remote monitoring. The management of side effects, diet, exercise, and questions related to treatment can be carried out anytime and anywhere using a mobile phone, and patients with cancer can have continuous personalized care.

Second, the mobile care systems can continuously provide both offline and online management services, and the partnership between the service provider and the patient can be strengthened. These systems can maintain the quality of ongoing daily care for patients on long-term chemotherapy or early chemotherapy.

Third, the mobile care system can provide accurate information based on clinical and professional knowledge. Patients can easily access various information related to cancer. Thus, health experts can provide real-time information based on experience.

Fourth, the possibility of online management of cancer patients with a mobile app was demonstrated in this study. Since the population undergoing cancer treatment is on the increase, the system should be enhanced by improving the quality of contents and user interface, which will also motivate patients to achieve a better quality of life.

### Limitations

This study has several limitations. First, the standardized methodologies related to the development such as biodesign process were not applied [38]. Thus, it may be difficult for others to benchmark our development process. Second, the study did not include a control group for comparison. This could limit the interpretation of the results from the outcome. Third, raw data as logs from apps were not available. These data could have made it possible to analyze details of patients’ behaviors with the app and devices. Fourth, we did not use standardized questions for the usability survey. Although satisfaction is a very important factor for usability, it does not solely represent it. Lastly, we did not describe values of specific clinical measures because they were not within the scope of this paper. This information would be available in future studies with specific knowledge of each topic: rehabilitation, chemotherapy, and nutrition.

### Conclusions

Through this study, we successfully developed and validated a multidisciplinary mobile care system that can provide health education and self-management features to improve clinical measures for patients with advanced gastrointestinal cancer. The system showed a high rate of program completion by patients with good satisfaction.

## Abbreviations

BFI-K: Brief Fatigue Inventory–Korean
EORTC QLQ-CR38: European Organization for Research and Treatment of Cancer Quality of Life Questionnaire–Colorectal module
EORTC QLQ-STO22: European Organization for Research and Treatment of Cancer Quality of Life Questionnaire–Gastric module
EORTC QLQ-30: European Organization for Research and Treatment of Cancer Quality of Life Questionnaire
IPAQ-SF: International Physical Activity Questionnaire–Short Form
LARS: Low Anterior Resection Syndrome
PG-SGA: Scored Patient-Generated Subjective Global Assessment
PRO-CTCAE: Patient-Reported Outcomes version of the Common Terminology Criteria for Adverse Events
